# Left Lower Abdominal Pain as an Initial Symptom of Multiple Myeloma

**DOI:** 10.7759/cureus.20652

**Published:** 2021-12-23

**Authors:** Fumiko Yamane, Ryuichi Ohta, Chiaki Sano

**Affiliations:** 1 Community Care, Unnan City Hospital, Unnan, JPN; 2 Community Medicine Management, Faculty of Medicine, Shimane University, Izumo, JPN

**Keywords:** rural hospital, vertebral lesion, back pain, abdominal pain, multiple myeloma

## Abstract

Multiple myeloma can present with various general symptoms such as fever, fatigue, and night sweats. Bone pain can occur in the pelvic and vertebral bones. However, there are a few reports of abdominal pain as an initial symptom of multiple myeloma. Here, we report the case of a 73-year-old male patient with a chief complaint of acute left lower abdominal pain. The abdominal physical findings were unremarkable. The pain was considered as referred pain, but there was no pain in response to a knock on the back. Further investigation using enhanced abdominal CT revealed a lesion in the left vertebral arch of the 10th thoracic vertebra. Bone marrow biopsy led to a diagnosis of IgA-type multiple myeloma. This case shows that abdominal pain could indicate vertebral lesions, and even without back pain, the condition of the vertebral arches should be investigated.

## Introduction

Abdominal pain is a common symptom in outpatient and emergency departments and shows various presentations. Abdominal pain can be diagnosed by physicians (approximately 50%), and many patients with abdominal pain may not be diagnosed and cured during the natural course [1]. Abdominal pain is classified into three categories: visceral, parietal, and referred pain [2]. The differentiation of these pains is critical for the diagnosis of abdominal pain [2]. Referred pain is difficult to diagnose because it can occur in various locations due to various diseases [3]. To diagnose the referred pain, precise medical history and physical examination are needed.

A frequently occurring referred pain is abdominal pain with lower back pain and this is commonly seen with vertebral lesions, such as compression fractures among older patients [4-5]. Diagnosis can be performed based on the physical findings of thoracic or lumbar vertebral tenderness [4-5]. Most cases of this type of pain can be benign and are missed because of their self-limited features [6]. However, bone lesions may also rarely cause referred pain in the abdomen. One such disease is multiple myeloma, which can be critical and should be diagnosed [7]. Previous research has shown that the initial presentation of multiple myeloma with a bone lesion is bone pain; therefore, back pain among older patients with hypercalcemia, anemia, bone lesions, and renal abnormalities (calcium elevation, renal insufficiency, anemia, and bone abnormalities -- CRAB symptoms) can be diagnosed with multiple myeloma [7]. However, if there is no bone or back pain without renal dysfunction, the diagnosis can be challenging. We report the case of a male patient with acute abdominal pain without back pain who was diagnosed with multiple myeloma. The purpose of this case report was to demonstrate the importance of history taking and physical examination of abdominal pain and the presence of multiple myeloma of the vertebra without lower back pain.

## Case presentation

A 73-year-old man visited a community hospital with the chief complaint of a one-month dull and persistent pain in the lower left abdomen. One week before coming to the hospital, he noticed pain at night while sleeping. The pain worsened while lying supine and improved with standing. Two days before he visited our department, the abdominal pain worsened, and he visited the emergency department. He was diagnosed with functional back pain. He did not have other symptoms such as fever, appetite loss, weight loss, night sweats, joint pain, and back pain. His daily activities were normal, and his medical history included hypertension and dyslipidemia. His medications included amlodipine and simvastatin. The initial vital signs were blood pressure of 122/75 mmHg, regular pulse rate of 93/min, body temperature of 36.2°C, and oxygen saturation of 96% on room air. The physical abdominal findings included normal bowel sound, soft and flat abdomen, and tenderness in the lower left abdominal quadrant with deep palpation without any rash. Laboratory data showed a white blood cell of 3500/μL, hemoglobin (Hb) of 10.3 g/dL, and platelet count of 18×106/μL without hypoalbuminemia, hyperproteinemia, or proteinuria (Table 1).

**Table 1:** Laboratory values of the patient (day 1). MCV, mean corpuscular volume; AST, aspartate aminotransferase; ALT, alanine aminotransferase; γ-GTP, γ-glutamyl transpeptidase; LDH, lactate dehydrogenase; BUN, blood urea nitrogen; eGFR, estimated glomerular filtration rate; ALP, alkaline phosphatase

Maker	Level	Reference
White blood cell	3500	3.5–9.1 × 10^3/μL
Neutrophil	51.8	44.0–72.0%
Lymphocyte	31.0	18.0–59.0%
Monocyte	13.1	0.0–12.0%
Eosinophil	3.4	0.0–10.0%
Basophil	0.7	0.0–3.0%
Red blood cell	3150000	3.76–5.50 × 10^6/μ
Hemoglobin	10.3	11.3–15.2 g/dL
Hematocrit	30.8	33.4–44.9%
MCV	97.7	79.0–100.0 fl
Platelet	180000	13.0–36.9 × 10^4/μL
Total protein	8.0	6.5–8.3 g/dL
Albumin	4.0	3.8–5.3 g/dL
Total bilirubin	0.5	0.2–1.2 mg/dL
AST	22	8–38 IU/l
ALT	20	4–43 IU/l
ALP	151	106–322 U/L
γ-GTP	23	<48 IU/l
LDH	186	121–245 U/L
BUN	15.3	8–20 mg/dL
Creatinine	0.73	0.47–0.49 mg/dL
Serum Na	141	135–150 mEq/l
Serum K	44	3.5–5.3 mEq/l
Serum Cl	103	98–110 mEq/l
Serum Ca	10.0	8.8–10.2 mg/dL
Serum P	3.6	0.2–1.2 mg/dL
Serum Mg	2.1	1.8–2.3 mg/dL
eGFR	77.5	>60.0 mL/min/1.73m2
Urinalysis		
White blood cell	(-)	
Protein	(-)	
Glucose	(-)	
Occult hematuria	(-)	
Urinary protein amount	0.05	g/1.73m^3^

**Figure 1:**
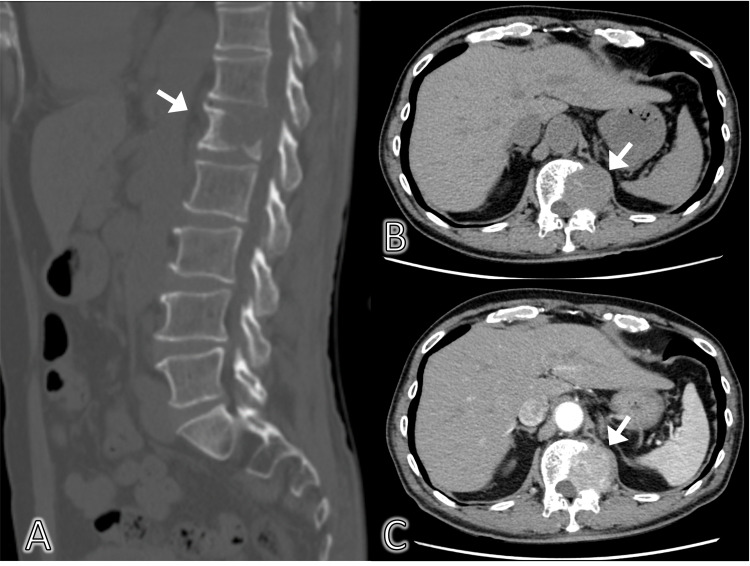
Abdominal CT (A. Sagittal plane, B. Coronal plain, C. Coronal plain with contrast). The osteolytic lesion in the first lumbar vertebra invading the left trabeculae on plain and enhanced CT.

The abdominal ultrasound did not show any abnormalities, such as ascites, intestinal dilatation, or any mass. We considered the possibility of referred pain in the left quadrant. Therefore, we performed abdominal CT to identify any impinging lesion on the path of the intercostal nerve on the left quadrant. CT and enhanced CT revealed a mass lesion on the left side of the first lumbar vertebral body invading the left trabeculae (Figure 1).

**Figure 2:**
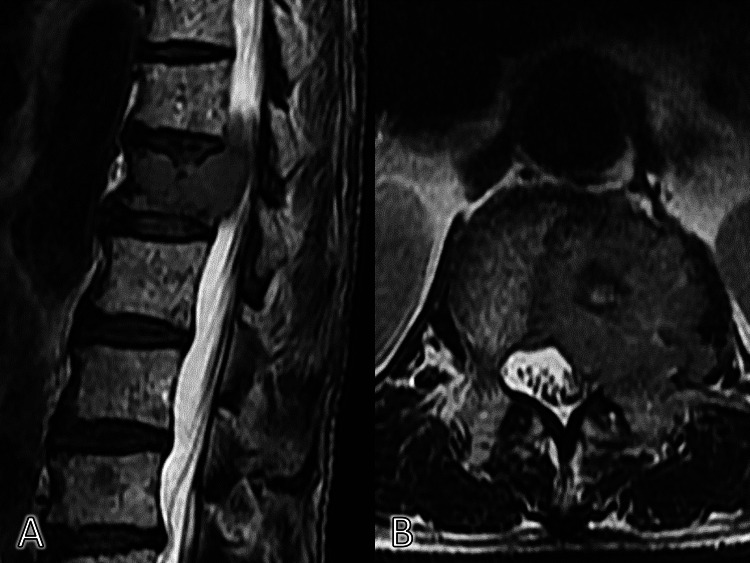
MRI of the vertebrae (A. Sagittal plane, B. Coronal plane). The osteolytic lesion in the first lumbar vertebra invading the left trabeculae on MRI.

Furthermore, additional MRI showed that the mass invaded the left intervertebral foramen, which could cause referred pain in the left quadrant of the abdomen (Figure 2).

**Figure 3:**
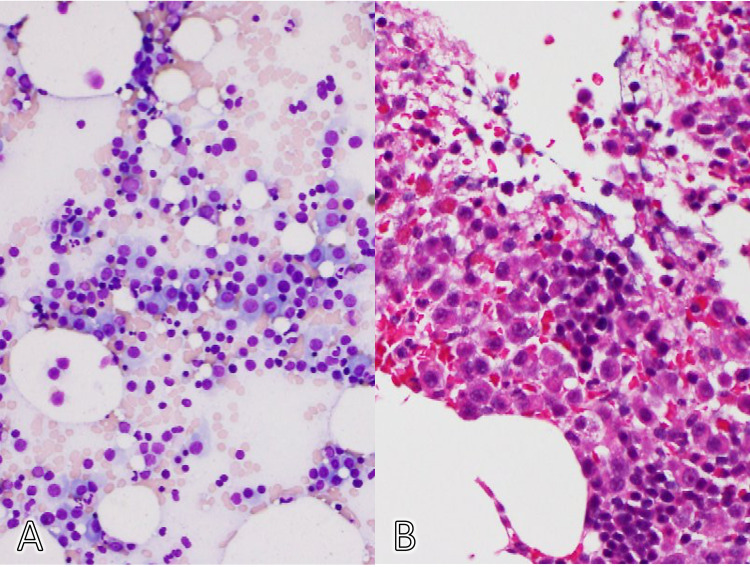
Histological analysis of the bone marrow biopsy. A. May Grunwald Giemsa stain, B. Hematoxylin-eosin stain

Based on the clinical findings, we suspected multiple myeloma and performed a bone marrow biopsy. Bone marrow biopsy showed a plasma cell neoplasm with 26.0% of cells being plasma cells (Figure 3).

**Figure 4:**
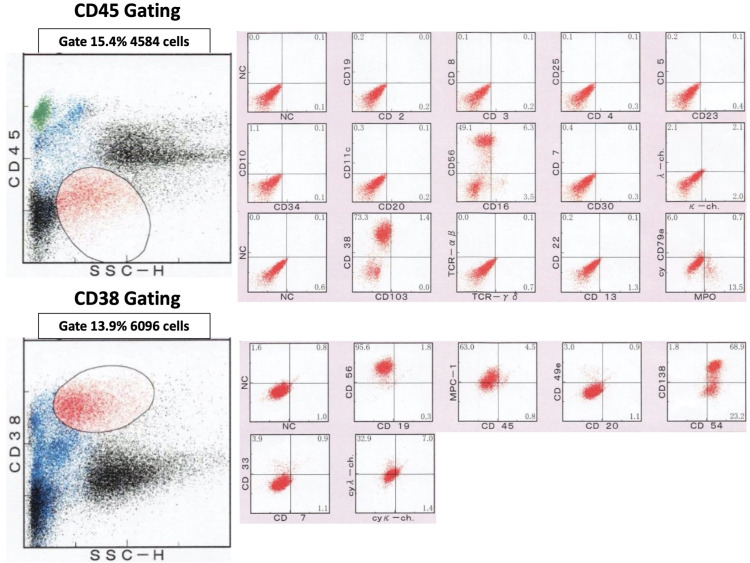
Flow cytometric analysis of bone marrow biopsy. Flow cytometry of bone marrow-derived CD38+ and CD45+ cells showed that the plasma neoplasm was CD56+, CD38+, CD138+, and MPC-1+.

We additionally checked erythrocyte sedimentation rate (ESR) and plasma immunoglobulin, resulting in an ESR of 97 mm/h (normal range: <15 mm/h), immunoglobulin (IgG) of 575 mg/dL (normal range: 700-1600 mg/dL), immunoglobulin A (IgA) of 2439 mg/dL (normal range: 80-350 mg/dL), and immunoglobulin M (IgM) of 11 mg/dL (normal range: 40-250 mg/dL). Plasma and urine investigations for Bence-Jones protein were negative. The serum immunoelectrophoresis showed the IgG and IgM had decreased and IgA had increased. The κ/λ ratio of the free light chain was 0.43 (normal range: 0.26-1.65). Flow cytometry of bone marrow-derived CD38+ and CD45+ cells showed that the plasma neoplasm was CD56+, CD38+, CD138+, and MPC-1+ (Figure 4).

Chromosomal analysis (G-band) revealed abnormalities of 45, X, -Y, +5, +6, +7, -8, +9, +11, -13, and +21. We diagnosed the patient with IgA-type multiple myeloma and referred the patient to a hematologist for specific treatment at a university hospital.

## Discussion

This case shows the rare presentation of multiple myeloma as abdominal pain without back pain and the importance of a broad differential diagnosis of referred abdominal pain. Occupying lesions of the lumbar vertebrae due to malignancy may not cause back and bone pain; they may only cause referred pain to the abdominal wall. Therefore, an absence of back pain may not rule out progressive bone diseases of the back.

Abdominal pain with multiple myeloma is rare but alarming. Abdominal pain can be investigated by appropriate history taking and physical examination. Non-localized abdominal pain without tenderness on the location of the pain can be considered as referred pain from the root or nerves related to the abdomen [3]. Innervation of the abdomen is mainly from the intercostal nerves of the spinal cord [8]. The referred pain from the intercostal nerves can occur due to compression or invasion from the outside, such as vertebral hernia, compression fracture, and malignancy [4-5, 9-10]. The most common etiology is compression fracture, especially among older people [4-5]. A compression fracture compresses the intercostal nerve laterally and vertically, which can cause general pain in the back to the abdomen, similar to a vertebral disc hernia. The differentiation between the two can be the onset of symptoms. A compression fracture can be acute onset [4-5], while disc herniation can be subacute to chronic similar to malignancy [10]. However, in this case, the pain in the abdomen occurred acutely and progressed gradually. Furthermore, this patient did not have back pain, tenderness on the back, or any alarming symptoms. This clinical course can be atypical regarding the presentation of multiple myeloma, which can be related to the location of malignancy in vertebrae [7, 11-12].

The quality and quantity of referred pain by mass lesions of the vertebral arches can differ depending on the location of the mass. Masses that are located in the vertebral body and invade the bone cortex can trigger inflammation in surrounding tissues, which can cause pain in the location [13]. On the other hand, masses that are located surrounding vertebral joints or facet joints can initially trigger musculoskeletal and neurogenic pain by specific movements and postures, which can include both [14-15]. Neurogenic pain can be referred to the abdomen and inguinal parts [14-15]. In this case, the mass was located between the right pedicle and the body of the vertebra, which could affect the presentation of symptoms of no back pain. The lesion of the pedicle may incur pain but can involve the intercostal nerves, which can induce pain in the abdomen. This presentation of pain can occur in patients with compression fractures in older people [16]. However, in this case, the patient did not have any trauma or risk related to osteoporosis. This case shows the atypical presentation of bone tumors, including multiple myeloma, and the importance of precisely considering the quality of pain in the diagnosis of abdominal pain.

## Conclusions

Referred pain can be difficult to diagnose without localized pain on the back among patients with multiple myeloma. Abdominal pain has various differential diagnoses, causing confusion among clinicians. Multiple myeloma can cause bone lesions only causing abdominal pain without CRAB symptoms. Referred pain in the abdomen should alert clinicians to check the backbone to ensure that malignancies of the bone such as multiple myeloma are not missed for prompt diagnosis and treatments.
